# Clinical Evaluation of Three Surgical Modalities in the Treatment of Peri-Implantitis: A Randomized Controlled Clinical Trial

**DOI:** 10.3390/jcm8070966

**Published:** 2019-07-03

**Authors:** Selena Toma, Michel C. Brecx, Jerome F. Lasserre

**Affiliations:** 1 Department of Periodontology, Université Catholique de Louvain (UCL)—Cliniques Universitaires Saint Luc, Brussels 1200, Belgium; 2 Institut de Recherche Expérimentale et Clinique (IREC), Pôle de Morphologie, Université Catholique de Louvain (UCL), Brussels 1200, Belgium

**Keywords:** biofilm, peri-implantitis, surgical treatment, titanium surfaces decontamination

## Abstract

Objectives: To compare the efficacy of three mechanical procedures for surgically treating peri-implantitis. Materials and Methods: In a randomized, prospective, parallel-group study, 47 patients with peri-implantitis were treated with (a) plastic curettes (*n* = 15 patients, 25 implants), (b) an air-abrasive device (Perio-Flow^®^, *n* = 16 patients,22 implants), or (c) a titanium brush (Ti-Brush^®^, *n* = 16 patients, 23 implants). Patients were assessed for the following measures at three timepoints (baseline, and three and six months after surgery): plaque index, bleeding on probing, gingival index, probing pocket depth (PPD), relative attachment level, and bone loss. Treatment outcome was considered successful when the implant was still present with PPD ≤ 5 mm, no bleeding on probing, and no further mean bone loss ≥ 0.5 mm. Results: A greater reduction of gingival index and PPD was observed in the titanium brush group than in the other groups at six months (*P* < 0.001). Relative attachment level decreased from baseline in each group at three months but was more marked in the titanium brush group (*P* < 0.001). At six months, there was less bone loss in the titanium brush group than in the plastic curette group (*P* < 0.001; linear mixed model and Kruskal–Wallis). A successful outcome was observed in 22% of implants in the plastic curette group, 27% in the Perio-Flow^®^ group, and 33% in the Ti-Brush^®^ group. Conclusions: The titanium brush and glycine air-polishing device were more effective than the other methods, but treatment success remained low. Combining mechanical procedures with antimicrobials and/or antibiotics might be a more effective strategy and warrants careful investigation.

## 1. Introduction

Dental implants are a common treatment after tooth loss. However, despite high survival rates of 96.33% eight years after initial placement [1], biological complications do occur [2], including peri-implantitis—which involves bone loss and bleeding on probing, with or without suppuration [3]—and peri-implant mucositis. The prevalence of peri-implantitis seems to reach 18.5% at patient level and 12.8% at implant level according to a recent meta-analysis [4]. Peri-implant mucositis, a prerequisite for peri-implantitis, has been estimated to affect 63.4% of patients (30.7% of implants) [5]. However, a review of the literature reveals little consensus on the prevalence of such infections, which may indicate a need for standardized diagnostic criteria and clear definition. When supportive maintenance therapy is not carried out regularly, peri-implant disease can lead to implant loss. Good oral hygiene—and greater access to implant sites for oral hygiene—lessen the frequency of peri-implantitis [6] and, thus, lessen marginal bone loss [7]. Moreover, plaque index is positively correlated with peri-implant disease [8].

Peri-implantitis, like periodontitis, is caused most of the time by bacteria that adhere to the surface of the implant and organize in biofilms that spread across its surface [9]. Therefore, periodontitis and peri-implantitis treatments share the same primary goal: removal of the biofilm [10]. Because of their similar pathophysiologies, protocols for treating periodontitis have been co-opted for peri-implant disease [11]. However, there is no agreement as yet on the best course of treatment for peri-implant diseases, despite the testing of multiple protocols [10]. Nonsurgical procedures with curettes failed to demonstrate a significant improvement in patients [11,12]. Although surgical procedures improved clinical parameters, simple mechanical debridement produced similar outcomes in comparison to complex and expensive treatments [10]. The Consensus report of the 7th European Workshop on Periodontology recognized also that the onset and progression of peri-implantitis may be influenced by iatrogenic factors such as “inadequate restoration–abutment seating, overcontouring of restorations or implant-malpositioning” [3]. Implant mal-position, design of the suprastructure, as well as non-controlled occlusal forces, bone compression due to the insertion of the implant and so many other factors could influence the apparition of marginal bone loss. However, studies examining the role of iatrogenic factors in the development of peri-implant diseases are still scarce [3].

Successful treatment of peri-implantitis may be elusive, as reliable treatment outcomes—in terms of reduced inflammation surrounding implants—are rarely reported [13]. The 8th European Workshop on Periodontology issued a consensus report stressing the need for strictly controlled randomized clinical trials that measure outcomes at six- and 12-month endpoints (at a minimum) to determine a standard of treatment for peri-implantitis [14]. In addition, the report called on practitioners to include in published studies the number of patients in whom peri-implantitis was resolved or a successful treatment outcome was obtained, defined as implant survival with probing pocket depth (PPD) ≤ 5 mm without bleeding or suppuration. These considerations motivated the present study.

The aim of this randomized controlled trial was to compare the clinical and radiological efficacy of plastic curettes, an air-abrasive device (Perio-Flow^®^) and a titanium brush (Ti-Brush^®^) during surgical treatment of patients with peri-implantitis.

## 2. Materials and Methods

### 2.1. Study Design

A prospective, single-blind, parallel group randomized, controlled trial with a 6-month follow-up was conducted to assess the clinical and radiological effects of three mechanical procedures for the surgical treatment of peri-implantitis: plastic curettes, a Perio-Flow^®^ device, and a Ti-Brush^®^. The study followed the Helsinki Declaration and was registered with the ethical committee of the Medical School of the Université Catholique de Louvain, Brussels, Belgium (2013/18MAR/122—No. B403201317011).

### 2.2. Participants

We recruited patients from the dental school of the Université Catholique de Louvain (Cliniques universitaires Saint-Luc), Brussels, Belgium. All participants were screened during periodontal consultations. We informed them about peri-implant disease and the aims and duration of the study and provided them a complete protocol. All participants signed an informed consent agreement after deciding to enroll. Recruitment, treatment, and follow-up were completed between August 2013 and July 2017. As proposed by the VII European Workshop on Periodontology, modified Consolidated Standards of Reporting Trials (CONSORT) guidelines were applied.

The required sample size per treatment group was *n* = 15 patients or implants, based on a 1 mm (standard deviation (SD) = 0.5 mm) reduction in PPD, a significance threshold of *P* = 0.05, and 90% power.

As suggested by Carcuac et al. [15], treatment success was defined as a PPD ≤ 5 mm, absence of bleeding or suppuration on probing at the implant site upon examination at six months, and no additional mean bone loss ≥ 0.5 mm between the baseline and six-month timepoints.

### 2.3. Inclusion and Exclusion Criteria

Inclusion criteria required that patients present with one or more implants showing signs of peri-implantitis with (a) bleeding or suppuration on probing; (b) PPD ≥ 5 mm; (c) complete immobility of the implant; (d) radiographic evidence of bone loss ≥ 2 mm or resulting in exposure of two or more implant threads for systems with visible implant threads; (e) no occlusal overload (checked by occlusion papers); (f) treated periodontitis with Dutch periodontal screening index ≤ 2; (g) no systemic disease or treatment, such as bisphosphonates, diabetes type 1 (A1c < 7%), or inflammatory diseases that might influence treatment or outcome; and (h) no presurgical antibiotic (local or systemic) or oral antiseptic for three months. To be included, patients had to meet all these criteria.

Individuals with poor general health, need for antibiotic prophylaxis before the surgical procedure, allergy to penicillin, or current pregnancy or lactation, were excluded from participation.

Patients were also excluded if the design of the superstructure was not appropriate for peri-implantitis evaluation due to a limited access to the peri-implant pocket.

A total of 47 surgically-treated participants with peri-implantitis (a total of 70 implants) were allocated to one of the three treatment groups for decontamination using a randomized computer-generated list (SPSS, PASW Statistics for Windows, version 18.0, SPSS Inc, Chicago, IL, USA) as follows (see Table 1): plastic curette (control group; *n* = 15 patients, 25 implants), Perio-Flow^®^ (*n* = 16 patients, 22 implants), and Ti-Brush^®^ (*n* = 16 patients, 23 implants). This list was generated before initiation of the study by the principal investigator (ST).

### 2.4. Baseline Clinical Examination

The following baseline clinical parameters were recorded using a manually calibrated periodontal probe with a constant force of 0.2 N (20 g) (WHO DB765R, Aesculap, Tuttingen, Germany): full-mouth plaque score and full-mouth bleeding score, excluding the wisdom teeth. One investigator clinician (ST) measured each variable (PPD, bleeding on probing, plaque index, gingival index, and relative attachment level (RAL)) at six locations per implant: mesiovestibular, midvestibular, distovestibular, mesiolingual, midlingual and distolingual. No prostheses were removed during the assessments.

### 2.5. Radiographic Examination

Intra-oral radiographs were collected for each implant at baseline and six months using the long cone paralleling technique with phosphor plates (74321; Durr Dental AG, Bietigheim-Bissingen, Germany) and a sensor holder (Eggen-holder/Super-Bite blocks; Kerr Dental, Orange, CA, USA). Radiographs were analyzed using Sidexis XG 2.52 (Sirona Dental Systems GmbH, Germany). We used the implant–crown interface as the reference level and measured the distance (mm) between this and the bone at baseline and six months for each implant on the mesial and distal aspects. In order to avoid any bias, one investigator (DG), blinded to treatment group, used the same X-ray device for all pre- and postoperative radiographs, following a standardized procedure (same angulation by using a silicon jig). We selected five radiographs of two implants with bone loss ≥ 2 mm on at least one aspect for intra-examiner calibration. Mean values were calculated twice, with 48 h separating evaluations. Measurements within 0.5 mm more than 90% of the time were considered acceptable calibrations. To assess bone level changes from baseline we also collected intra-oral radiographs at six months.

### 2.6. Oral Hygiene Program

Two weeks prior to surgery, we provided individualized oral hygiene instructions and professional supragingival/mucosal cleaning for each patient, using a rubber cup with polishing paste (Nupro^®^ Prophy Grip, Dentsply, Woodbridge, Ontario, Canada). A surgery consultation was given three and six months after treatment, each ending with an oral hygiene reminder and professional supragingival/mucosal cleaning.

### 2.7. Surgical Treatments

All surgeries were performed under local anesthesia. Where possible, screw-retained suprastructures were removed on the day of surgery and replaced at the end afterwards. Intrasulcular incisions were performed at each implant site, followed by elevation of full-thickness flaps on the vestibular and lingual aspects. Vertical releasing incisions were also added if necessary. No antimicrobials or antibiotics were used during surgery.

**Figure 1:**
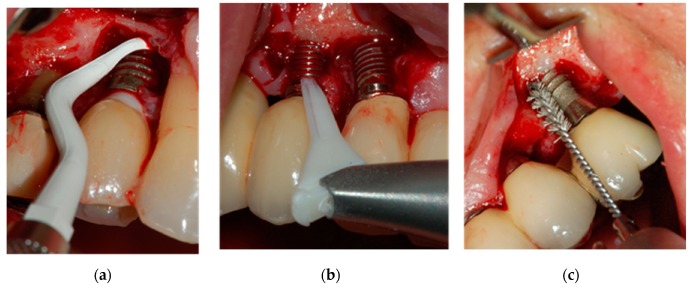
Surgical treatment of peri-implantitis with a plastic curette (**a**), Perio-Flow^®^ device (**b**), and Ti-Brush^®^ (**c**). Images were taken after the full-thickness elevation of a mucoperiosteal flap and granulation tissue removal.

In the plastic curette group (Figure 1), granulation tissue was entirely removed from the osseous defect and a plastic curette was used to scale the surface of the implant (Gracey curette, Implacare kit assort IMPHDL 6 TIP6, Hu-Friedy). The defect was then irrigated with sterile saline (20 mL, 20 s).

Surgical treatment for the Perio-Flow^®^ group (Figure 1) was previously described in Toma et al. [13]. In brief, granulation tissue was removed before using the Perio-Flow^®^ device (Perio-Flow Handy, Perio-Flow nozzle; EMS Medical, Nyon, Switzerland). Amino acid glycine powder (Air-Flow Perio Powder, EMS Medical) was projected using a specific nozzle placed parallel to the implant surface at each aspect, from coronal to apical, with 5 s of noncontact-mode circular motion, followed by sterile saline irrigation. To limit powder accumulation and to reduce emphysema complications, a high-speed evacuation system was placed 50 mm from the air-abrasive nozzle.

In the group treated with the Ti-Brush^®^ (Straumann, Basel, Switzerland; Figure 1), implants were cleaned using the brush for approximately 30 s. The brush consisted of a stainless-steel shaft and titanium bristles mounted on a surgical handpiece (Bien-Air Medical Technologies, Bienne, Switzerland) oscillating at low speed (900 oscillations per minute max) clockwise and counterclockwise. The treatment site was irrigated and cooled with sterile saline (NaCl).

One surgeon (ST) performed all treatments. To ensure healing, mattress sutures (horizontal and vertical) were used to reposition the full-thickness flap in all groups.

### 2.8. Postoperative Care

No postoperative antibiotics were prescribed, but we instructed participants to take 3 g of paracetamol daily for 10 days and to rinse their mouths with a solution of chlorhexidine digluconate (0.2%; Corsodyl, GlaxoSmithKline Consumer Healthcare, Buhl, Germany) for 1 min, twice daily for 10 days. We removed the sutures 10 days after surgery. Patients were asked to avoid mechanical brushing on the treated region for one week. A control healing visit occurred one week postsurgery, during which sutures were removed and participants were recommended to conduct a soft mechanical cleaning, beginning with the second week after the intervention, using a very soft surgical dental brush with 0.15 mm bristles (Meridol^®^, GABA International AG, Therwil, Switzerland). Participants could progressively resume their usual oral hygiene measures from the third week after surgery. Three months and six months after surgery they returned for professional supragingival cleaning around the implant and teeth (performed by ST) and a reminder of oral hygiene technique.

### 2.9. Postoperative Evaluations at Three and Six Months

Using a periodontal probe calibrated to 0.2 N, the same clinical investigator (ST) assessed each clinical parameter at six aspects per implant, in all participants, at three and six months postoperatively. At control visits, professional supragingival and mucosal cleaning were performed in addition to reinforcement of oral hygiene. Adverse events were recorded throughout the study period.

### 2.10. Statistical Analysis

Clinical and radiological parameters recorded during the study period are expressed as means with standard deviations. PPD was the primary outcome and results were considered statistically significant at *α* = 0.05.

For secondary outcomes, the intragroup evolution and intergroup comparison during follow-up were evaluated for each parameter and compared to baseline using a linear mixed model, except for bone loss, for which a Kruskal–Wallis rank sum test was applied. We used the open source statistical software R for all statistical analyses. Bonferroni correction for multiple comparisons maintained an alpha level of 0.05. The level of significance was therefore obtained if *P* < 0.008.

Means between groups were compared for treatment success and a multivariate logistic regression was conducted with all three treatments (plastic curette, Perio-Flow^®^, Ti-Brush^®^) to examine the effect of specific factors on treatment outcome (history of periodontitis, type of implant, bone level or tissue level, and screwed or cemented prosthesis).

## 3. Results

Across 47 surgically treated patients with peri-implantitis, 70 implants were decontaminated using either (a) plastic curettes (control group; *n* = 15 patients, 25 implants); (b) a Perio-Flow^®^ device (*n* = 16 patients, 22 implants); or (c) a Ti-Brush^®^ (*n* = 16 patients, 23 implants) (Table 1).

**Figure 2:**
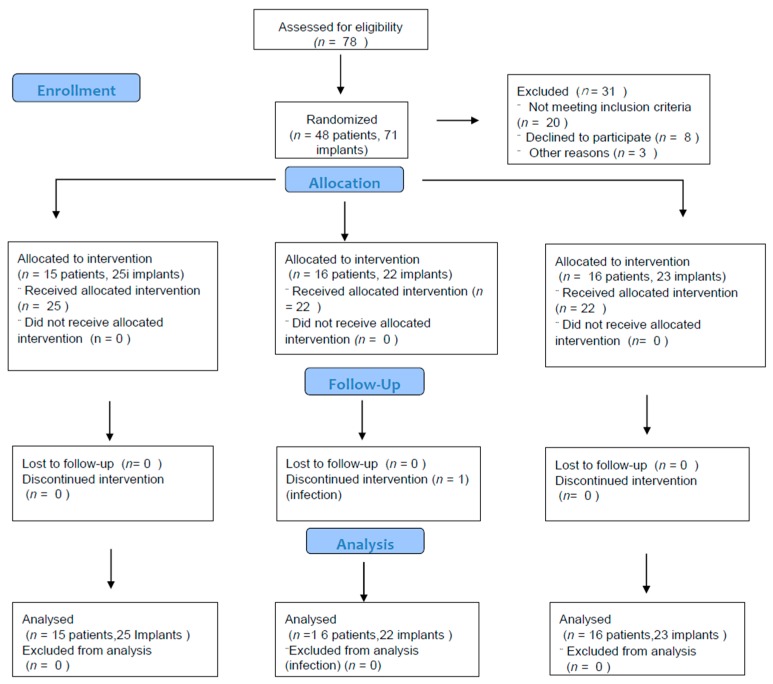
Study flowchart following the Consolidated Standards of Reporting Trials (CONSORT) guidelines.

Participants were randomized and allocated to one of three groups, as shown in the CONSORT flowchart (Figure 2). During the follow-up period, one case in the Perio-Flow^®^ group experienced complications (infection with persistence of suppuration and swelling). No other complications were observed (e.g., allergic reaction, abscess, emphysema). Participant characteristics are presented in Table 1.

The most common location for implants was the mandible: 86% in the plastic curette group, 72% in the Perio-Flow^®^ group, and 60% in the Ti-Brush^®^ group. All treated implants had a modified implant surface (100%), divided into a rough surface (22.8% titanium plasma spray) and microrough surface (77.2%). In all groups, fixed partial dental prostheses were the most frequent supraconstructions, and the mean time between implant placement and baseline across groups was 7.8 ± 2.36 years.

Periodontal status was either healthy or history of gingivitis in 16% of patients in the plastic curette group, 27% in the Perio-Flow^®^ group, and 18% in the Ti-Brush^®^ group. A history of moderate to severe periodontitis was recorded in 84% of patients in the plastic curette group, 73% in the Perio-Flow^®^ group, and 82% in the Ti-Brush^®^ group.

Of the implants in a fixed partial prosthesis, 76% were cemented in the plastic curette group, 65% in the Perio-Flow^®^ group, and 69% in the Ti-Brush^®^ group. The mean number (± SD) of remaining teeth per patient was 14.8 ± 8.5 in the plastic curette group, 16.8 ± 6.8 in the Perio-Flow^®^ group, and 17.3 in the Ti-Brush^®^ group. Patients had a mean of 2.4 implants each in the plastic curette group, 2.2 in the Perio-Flow^®^ group, and 2.1 in the Ti-Brush^®^ group. One implant in the Perio-Flow^®^ group was lost before the six-month follow-up due to acute infection.

### 3.1. Clinical Full-Mouth Parameters

In all groups, full-mouth plaque score was <30% at baseline and the mean decrease across all oral hygiene programs at the six-month follow-up visit was 25%. In parallel, the mean full-mouth bleeding score was also reduced from baseline in all groups at six months. These data were used to assess the compliance of the patient during the study period.

### 3.2. Implant Parameters

Plaque index remained low for the duration of the study (Table 2) and improved in all groups with a statistically significant effect of time, from baseline to 3 months (*P* < 0.001) and from baseline to six months (*P* < 0.001). In addition, at six months, a treatment effect revealed more improvement in plaque index for the titanium brush group than the plastic curette group (*P* < 0.001).

Similar decreases in gingival inflammation occurred in all groups. Bleeding on probing decreased from baseline in all three groups at three months (*P* < 0.001). At six months, the greatest reduction in gingival condition occurred in the Ti-Brush^®^ group. There was a significant reduction in gingival index between baseline and three months in all three modalities tested. No statistically significant between-group differences in gingival index were noted between three and six months (*P* > 0.05), but a more pronounced reduction was observed at six months in the Ti-Brush^®^ group compared to the plastic curette group (*P* < 0.01).

A reduction in PPD and RAL was observed in each group between baseline and three months (*P* < 0.001 for each group) and between three and six months (*P* < 0.001 for each group), with a greater reduction at six months in the Perio-Flow^®^ and Ti-Brush^®^ groups than in the plastic curettes group (*P* < 0.001 for each comparison) for both PPD and RAL (Table 2).

**Figure 3:**
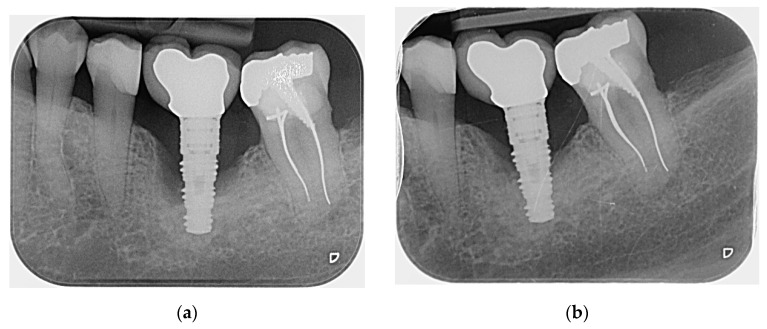
Radiographs of a peri-implant lesion before (**a**) and six months after (**b**) surgical treatment with the Ti-Brush^®^.

There was significantly less bone loss in the Ti-Brush^®^ than in the plastic curette groups at 6 months (*P* < 0.05; Table 2, Figure 3).

We found no significant differences in history of periodontitis or type of implants (bone level versus tissue level and screwed versus cemented prosthesis) between the three treatment groups (multivariate logistic regression (*P* < 0.05).

### 3.3. Treatment Outcome

Lastly, we examined resolution of peri-implantitis (i.e., successful treatment outcome), defined as implant survival with PPD ≤ 5 mm, no bleeding or suppuration on probing, and no additional bone loss ≥ 0.5 mm compared to baseline [15]. Across all treatment groups, success was achieved for 27% of all implants at six months. Specifically, 22% of implants in the plastic curette group, 29% in the Perio-Flow^®^ group and 33% in the Ti-Brush^®^ group (*P* < 0.05) had successful treatment outcomes.

## 4. Discussion

The purpose of this randomized controlled clinical trial was to compare the therapeutic efficacy of three decontamination procedures during surgical treatment of peri-implantitis. Antibiotics and chemical agents are frequently used with decontamination procedures, so the contribution of each aspect of the treatment needs to be examined separately. Experimental and clinical evaluations of decontamination protocols, including sterile saline, hydrogen peroxide and CO_2_ laser, have failed to demonstrate the superiority of any one method [16]. The conclusion of a Cochrane systematic review was that the complexity of the different methods makes it difficult to demonstrate reliable evidence for which is the most effective intervention for peri-implantitis [10]. Most of the protocols found in the literature are associated with resective and regenerative procedures. In addition, disease resolution, which is the primary goal of treatment, is rarely reported [11]. These considerations motivated the present study.

Nonsurgical debridement and surgical intervention are two commonly used regimens for treating peri-implant disease. Non-surgical treatment includes mechanical debridement and cleaning of implant surfaces. It is performed as a supportive therapy and can be effective in the treatment of peri-implant mucositis [11], but its effectiveness against peri-implantitis has not been demonstrated [17]. According to a consensus report, available evidence suggests that the outcome of nonsurgical treatment is unpredictable in cases of peri-implantitis [18].

We observed improvements in clinical parameters at three months, namely resolved or reduced inflammation (gingival index) and decreased mean PPD and RAL. These improvements were maintained for up to six months. As described by Shibli et al., a smaller PPD results in an environment less conducive to the proliferation of peri-implant pathogens, as well as better access for cleaning [19]. In this study, a successful management of moderate to severe peri-implantitis with a surgical protocol that included only mechanical decontamination was poorly achieved. The results obtained could be explained by the percentage of history of periodontitis in each group which is considered as a risk factor. However, a longer follow-up period is warranted to assess the long-term effects of the tested techniques. Among the three treatment modalities we examined, the Perio-Flow^®^ and Ti-Brush^®^ protocols were more effective than plastic curettes. These results are in accordance with previous work describing nonmetallic curettes as ineffective for removing bacteria, whereas air-powder abrasive, with amino acid glycine powder or sodium bicarbonate effectively cleans a range of titanium surfaces (from machined to rough and micro-rough surfaces) [20]. We observed treatment success in 27% of all implants, but success rates were highest in implants treated with the Ti-Brush^®^. This supports the observation by John et al. (2014) that the Ti-Brush^®^ removes plaque more effectively than steel curettes, and has a more gentle action on the implant surface [21]. 40% of the implants treated with the titanium brush (Ti-Brush^®^) were located in the maxilla compared to the other groups (14% for the plastic curette and 18% for the Perio-Flow^®^ group. According to Mameno et al., maxillary placement represents a higher risk of complication [22]. The results obtained in this study in the Ti-Brush^®^ could be underestimated. Furthermore, recent data demonstrated that treatment with plastic curettes, Perio-Flow^®^, Ti-Brush^®^ or implantoplasty does not disrupt the biocompatibility between titanium surfaces and osteoblasts [23]. Those findings, combined with the superior results we obtained with Perio-Flow^®^, indicate that Perio-Flow^®^ may be therapeutically more effective than other methods [23].

Our results indicate that, during surgical procedures, treating implant surfaces using plastic curettes should no longer be considered the gold-standard method. Evaluation of an air-abrasive device or a rotating titanium brush, in combination with systemic antibiotics, is necessary. Heitz-Mayfield et al. conducted a 12-month prospective study in which patients who received an antibiotic regimen comprising 500 mg amoxicillin and 400 mg metronidazole three times a day for the first seven days after surgery showed complete resolution of inflammation (no bleeding on probing) in 47% of 36 surgically treated implants, and 92% of these had stable crestal bone levels. However, even with the same antibiotics, not all studies report similar rates of disease resolution (e.g., 23% [24]) 12 months after open flap debridement when defining treatment success as a composite of shallow pockets, no bleeding on probing, and no further bone loss [24]. A recent study evaluating the effect of adjunctive systemic and local antimicrobial therapy in the surgical treatment of peri-implantitis demonstrated that local chlorhexidine application did not affect overall treatment outcome, and that modified implant surfaces were significantly less likely to result in treatment success [15]. By adding systemic antibiotics (750 mg amoxicillin twice daily for 10 days), the authors achieved a 58.8% success rate at 12 months with surface-modified implants, compared to a 16% success rate without amoxicillin. The authors concluded that the substantial influence of adjunctive systemic antibiotics depends on implant surface characteristics [15]. Another recent study found that adjunctive systemic azithromycin provided no clinical benefits at 12 months compared with open flap debridement alone [25]]. Randomized clinical trials should evaluate adjunctive use of systemic antibiotics in patients with systemic factors, such as those who smoke or have a history of periodontitis.

The 8th European Workshop on Periodontology stated that clinical research related to therapeutic approaches of peri-implantitis should report composite measures of disease resolution [14]. Following that recommendation, we evaluated success rate according to the guidelines of Carcuac and Derks [15], who defined the resolution of peri-implantitis as implant survival with PPD ≤ 5 mm, no bleeding or suppuration on probing, and no additional mean bone loss ≥ 0.5 mm relative to baseline. Another composite measure reported in the literature [26] defines a positive outcome as a mean reduction of PPD ≥ 0.5 mm with no bone loss relative to baseline. These differing conceptualizations of what constitutes a positive treatment outcome emphasize a need to establish consensus when reporting therapeutic outcome of peri-implantitis treatment. The most clinically applicable criterion and the most useful in everyday dental practice seems to be no further bone loss after surgery.

Here, we attempted to clearly define peri-implantitis, population, implant characteristics, interventions, and outcomes as proposed by Graziani and Figuero [27], and followed the CONSORT guidelines [28,29], in the interest of improving the specificity of peri-implant treatment research.

We attentively monitored plaque control and patient compliance during the follow-up visits at three and six months postoperatively. This monitoring was essential, as most participants had a history of periodontitis. According to Ramanauskaite et al. [30], although implant survival rate does not differ between patients with and without periodontitis, implant success rates (defined by the amount of marginal bone loss and incidence of peri-implantitis) were lower in patients with a history of periodontitis than in those without. Heitz-Mayfield et al. [31] investigated supportive peri-implant therapy after surgical treatment of peri-implantitis in a five-year survival and success clinical study. They demonstrated that the peri-implant conditions established after peri-implantitis surgery were maintained in most patients and implants after five years of regular supportive therapy, although peri-implantitis did recur in some patients, and others lost implants before final follow-up [31].

No bone substitute was used in the present study to manage bony defects after surgical debridement and decontamination; an adjunctive treatment with a bone xenograft seems to provide a more reliable outcome [32].

Most of the implants included in this study concerned partial edentulous patients restored with fixed prosthesis. Only 20% of the patients in each group were fully edentulous and restored with full arch removable prosthesis. A recent study observed that the type of support has a small but significant effect on implant prognosis. There was, furthermore, a tendency toward a greater incidence of complications for implants restored with removable dental prosthesis than for single crowns [33]. These observations seem to be not applicable in this study, nevertheless a strict follow-up protocol must be installed in patients presenting a complete removable prosthesis due to a possible greater incidence of complications.

Surgical malposition, non-controlled occlusal forces, bone compression during the insertion of the implant and so many other factors can influence the apparition of marginal bone loss [3]. The implant position and design of the suprastructure should be planned in order to facilitate access for self-performed oral hygiene and professionally administered plaque removal.

Nevertheless, even if a lot of risks factors and secondary etiological hypothesis exist, the biofilm theory remains the most accepted one and the trigger point of most of the peri-implantitis. Even in peri-implantitis cases where early marginal bone loss was influenced by surgical factors or prosthetic factors, the progression of the lesions is most of the time due to an inflammatory reaction caused by oral biofilm. Elimination of oral biofilm thus becomes a primary therapeutic goal. The elimination and decontamination of the titanium surface will allow a reduction of inflammation followed by a decrease in PPD and progressive bone loss. Further study must monitor more accurately surgical and prosthetic factors in order to avoid further biological complications.

This study is limited by a short evaluation period and a small sample number. Nevertheless, given the lack of randomized clinical trials evaluating only the mechanical treatment of peri-implantitis, the present results provide valuable information about a specific aspect of commonly used complex procedures and could serve to motivate longer and larger studies in the future.

## 5. Conclusions

In conclusion, this randomized controlled clinical trial involving three treatment modalities (plastic curettes, an air-abrasive device, and a titanium brush) showed that mechanical disinfection procedures effectively improved peri-implant disease-associated parameters over a short-term period. The titanium brush was more effective than the plastic curettes. A longer follow-up period is required for patients who displayed crestal bone stability six months postoperatively to confirm the longevity of the effects of our treatments. Lastly, complete resolution of the disease process, combining shallow pockets, no bleeding or suppuration on probing, and no additional bone loss from baseline, was difficult to achieve, even in the titanium brush group. This indicates that strict monitoring protocols should be implemented as soon as implant placement is considered.

## Figures and Tables

**Table 1:** Patient and implant characteristics for each group. Quantitative data are expressed as mean ± standard deviation.

	Plastic Curette	Perio-Flow^®^	Ti-Brush^®^
Age (years)	68.9 ± 15.8	67.5 ± 12.9	61.7 ± 13.4
Gender	77% F 23% M	90% F 10% M	81% F 19% M
Mandible	86%	72%	60%
Maxilla	14%	18%	40%
Micro-roughened surface	80.8%	78.9%	86.3%
Type of edentulism	76% partial 24% total	80% partial 20% total	77% partial 23% total
History of periodontitis	84%	73%	82%
Age of loading (years)	7.4 ± 1.9	8.8 ± 2.34	7.71 ± 2.12
Fixed partial prostheses	76% cemented 23% screwed	65% cemented 35% screwed	69% cemented 31% screwed
Remaining teeth/patient	14.8 ± 8.5	16.8 ± 6.8	17.3 ± 4.1
Number of implants/patient	2.4 ± 1.8	2.2 ± 2.2	2.1 ± 1.7

**Table 2:** Clinical and radiological data (mean ± standard deviation) of implants at baseline, and at 3 and 6 months postoperatively.

	Baseline	3 Months	6 Months
Plaque Index			
Plastic curette	1.33 ± 0.88	0.84 ± 0.43 ^†^	0.51 ± 0.54 ^†^
Perio-flow^®^	1.08 ± 0.57	0.89 ± 0.35 ^†^	0.45 ± 0.67 ^†^
Ti-Brush^®^	1.12 ± 0.44	0.78 ± 0.27 ^†^	0.30 ± 0.23 ^†,^*
Bleeding on Probing (%)			
Plastic curette	54 ± 4.4	21 ± 2.4 ^†^	29 ± 3.4 ^†^
Perio-flow^®^	59 ± 5.2	18 ± 4.2 ^†^	23 ± 2.3 ^†^
Ti-Brush^®^	62 ± 4.7	19 ± 5.1 ^†^	16 ± 3.7 ^†,^*
Gingival Index			
Plastic curette	1.55 ± 0.42	0.74 ± 0.46 ^†^	0.64 ± 0.37 ^†^
Perio-flow^®^	1.47 ± 0.37	0.89 ± 0.38 ^†^	0.51 ± 0.59 ^†^
Ti-Brush^®^	1.58 ± 0.45	0.76 ± 0.49 ^†^	0.44 ± 0.39 ^†,^*
Probing Pocket Depth (mm)			
Plastic curette	7.11 ± 1.15	5.54 ± 0.23 ^†^	5.44 ± 0.69 ^†^
Perio-flow^®^	6.94 ± 1.29	5.76 ± 0.34 ^†^	4.71 ± 1.24 ^†,^*
Ti-Brush^®^	6.45 ± 1.87	4.76 ± 0.21 ^†^	3.98 ± 1.43 ^†,^*
Relative Attachment Level (mm)			
Plastic curette	7.49 ± 1.49	6.38 ± 1.62 ^†^	5.82 ± 1.47 ^†^
Perio-flow^®^	6.94 ± 1.22	5.49 ± 1.57 ^†^	4.75 ± 1.38 ^†,^*
Ti-Brush^®^	7.03 ± 1.35	5.73 ± 1.55 ^†^	4.68 ± 1.32 ^†,^*
Bone Loss (mm)			
Plastic curette	6.49 ± 1.98	NE	5.99 ± 1.78
Perio-flow^®^	7.34 ± 1.29	NE	6.44 ± 1.46 ^†^
Ti-Brush^®^	7.09 ± 1.23	NE	5.88 ± 1.3 ^†,^*

^†^ Significantly different from baseline (intragroup comparison) (*P* < 0.001, linear mixed model with post hoc correction). * Significantly different from corresponding value in the other groups (intergroup comparison) (*P* < 0.001, linear mixed model with post hoc correction and Kruskall–Wallis sum rank test for bone loss).
